# Codivergence and multiple host species use by fig wasp populations of the *Ficus *pollination mutualism

**DOI:** 10.1186/1471-2148-12-1

**Published:** 2012-01-03

**Authors:** Michael J McLeish, Simon van Noort

**Affiliations:** 1 Department of Botany and Zoology, DST-NRF Centre of Excellence for Invasion Biology, Stellenbosch University, Private Bag X1, Matieland, 7602, South Africa; 2 Natural History Department, Iziko South African Museum, PO Box 61, Cape Town, 8000, South Africa; 3 Department of Zoology, University of Cape Town, Private Bag, Rondebosch, 7701, South Africa

## Abstract

**Background:**

The interaction between insects and plants takes myriad forms in the generation of spectacular diversity. In this association a species host range is fundamental and often measured using an estimate of phylogenetic concordance between species. Pollinating fig wasps display extreme host species specificity, but the intraspecific variation in empirical accounts of host affiliation has previously been underestimated. In this investigation, lineage delimitation and codiversification tests are used to generate and discuss hypotheses elucidating on pollinating fig wasp associations with *Ficus*.

**Results:**

Statistical parsimony and AMOVA revealed deep divergences at the *COI *locus within several pollinating fig wasp species that persist on the same host *Ficus *species. Changes in branching patterns estimated using the generalized mixed Yule coalescent test indicated lineage duplication on the same *Ficus *species. Conversely, *Elisabethiella *and *Alfonsiella *fig wasp species are able to reproduce on multiple, but closely related host fig species. Tree reconciliation tests indicate significant codiversification as well as significant incongruence between fig wasp and *Ficus *phylogenies.

**Conclusions:**

The findings demonstrate more relaxed pollinating fig wasp host specificity than previously appreciated. Evolutionarily conservative host associations have been tempered by horizontal transfer and lineage duplication among closely related *Ficus *species. Independent and asynchronistic diversification of pollinating fig wasps is best explained by a combination of both sympatric and allopatric models of speciation. Pollinator host preference constraints permit reproduction on closely related *Ficus *species, but uncertainty of the frequency and duration of these associations requires better resolution.

## Background

Several lines of theory have been proposed to account for the enormous diversity of phytophagous insects. Diversification conceivably ensues by ecological opportunity and adaptation to the exploitation of previously unattainable resources [1,2]; by restricted gene flow through allopatric means [3,4]; and disruptive selection and sympatric speciation [5,6]. In order to discern among potential mechanisms driving speciation, both historical pattern and ecological scale processes are important to consider [7-10]. Comparative phylogenetic approaches that test congruence between host and associate populations can contribute to greater resolution in unravelling ecological scale processes [11-14]. Here we interpret the codiversification between *Ficus *host species and populations of a group of African fig wasp pollinator species.

No single adaptive hypothesis is yet to explain the conditions determining the origin, maintenance, and extinction of insect-plant mutualisms [15] and the overwhelming amount of literature surrounding the field has led to periodic reassessment of central concepts [2,16,17]. Pollination mutualisms are iconic examples of highly specialized interactions [18-20]. Fig trees (Moraceae: *Ficus*) are singularly dependent on pollination by fig wasps (Chalcidoidea: Agaonidae) that in turn are dependent on the fig 'fruit' for reproduction [18,21]. Pollinating fig wasps show remarkable conservatism in host *Ficus *preference having concordant and ancient associations in the vicinity of 60 Myr [22,23]. Correlated evolution between figs and fig wasps is supported by numerous examples [18,24]. These include respiratory adaptation to fluid-filled figs [25] and head and mouthpart adaptation to the receptacle ostiole [26]. The presence of close phylogenetic correspondence within the mutualism has been presented in previous work [22,27-30] and strongly supports the hypothesis of a historically conservative interaction. The causal mechanisms supporting the maintenance of extremely conserved interactions remain unclear.

The estimation of the strength of these evolutionarily conserved interactions has come about through testing phylogenetic congruence at the species level and above. The presence of strict-sense cospeciation between one pollinator species and one *Ficus *species remain an outstanding case in insect-plant interactions, albeit greater scrutiny over the last decade has revealed species complexes and multiple pollinator species present on single host species [8,13,31]. From an empirical point of view, tests of phylogenetic correspondence that seek to estimate the level of 'cospeciation' [32] can in theory indicate up to four codiversification scenarios [33]. Thus, the taxonomic scale at which tests of congruence are conducted can influence interpretation of the cospeciation pattern in uncovering process. Analyses of congruence among distantly related taxonomic subdivisions in poorly sampled clades can bias interpretation in favour of 'cospeciation' [8]. Jackson and colleagues [9] recently demonstrated that the level of resolution of genetic variation can bias the interpretation of host species associations. Earlier work on the fig-fig wasp mutualism [26,28] tended to be conducted at the level of genus and species. Fig wasp host fidelity is perhaps most strong at the genus/host section level [8,22,23,26,34]. Hypotheses explaining discordance in phylogenetic congruence implicate independent speciation, extinction, lineage duplication of either host or associate as well as horizontal transfer between host species.

Recent work has highlighted uncertainties concerning taxonomic classification and difficulty in understanding the phylogenetic associations between African pollinator genera and *Ficus *section *Galoglychia *[35]. In this investigation, we test how independent diversification of agaonid pollinators is from host species of *Galoglychia*. Global measures of fit and reconciliation approaches are used to test phylogenetic congruence between pollinator fig wasp populations and *Ficus *species. Fig wasp within-species genetic variation at the cytochrome oxsidase one (*COI*) locus is assessed using statistical parsimony, AMOVA, and diversification modelling. Alternative pollinator topologies inferred with mitochondrial (mtDNA) and nuclear gene sequences are explored using likelihood ratio tests. The results were optimised over a set of hypotheses and are best characterised by pollinator populations reproducing on multiple *Ficus *species.

## Results

### Phylogenetic inference

Our Bayesian and parsimony bootstrap consensus inferences based on *EF-1α, COI*, and *Cytb *produced the same topology (Additional file 1). The Markov chain reached apparent stationarity after 2.0 × 10^6 ^generations and the last 10.0 × 10^6 ^generations of 20.0 × 10^6 ^generations were used to generate the pollinator and *Ficus *consensus phylogenies. Posterior probabilities (PP) for all genera were well resolved and well supported (PP = 100). Bootstrap support for the genus *Elisabethiella *was poor (47) as was the support for the bifurcations of genera especially for the *Courtella/Alfonsiella *node (PP = 83; bootstrap = 62). This topology was in general agreement with a phylogeny of Machado and colleagues [36] that was based on *COI *showing *Courtella *and *Elisabethiella *as sister-clades. However, the majority of other cases support a topology where *Elisabethiella *is sister-clade to *Alfonsiella *though topology outside this relationship is inconsistent [37] and pollinator body length compatibility with syconium size measurements supports this hypothesis as well [38]. Therefore, we used the harmonic means of four separate Bayesian runs to generate Bayes Factors [39] that compared constrained topologies of each case. The results were inconclusive (BF ~ 0.07) of one topology over the other so we used both in the reconciliation tests. We elected to use the *Elisabethiella *and *Alfonsiella *sister-genera inference for congruence testing as most studies support this topology and pilot analyses showed negligible differences between the two.

The *Ficus *phylogenetic inferences (Additional file 2) generated by Bayesian and parsimony approaches had the same topologies and are in general agreement with previous work [38]. The subsections *Chlamydodorae, Platyphylla*, and *Caulocarpae *form well-supported clades in the Bayesian inference (posterior probabilities > 99). The former two subsections show bootstrap support of 97 & 73 respectively and more equivocal support (52) for the *Caulocarpae *clade. Subsections *Urostigma *and *Sycomorus *were constrained to outgroup taxa. Singleton representatives of subsections *Crassicostae *and *Galoglychia *are consistent with the topology of previous work [38]. Due to the placement of *F. ilicina *(Sonder) Miq., the resulting polyphyly of *Chlamydodorae *necessitates future revision.

### Within-lineage delimitation

**Figure 1:**
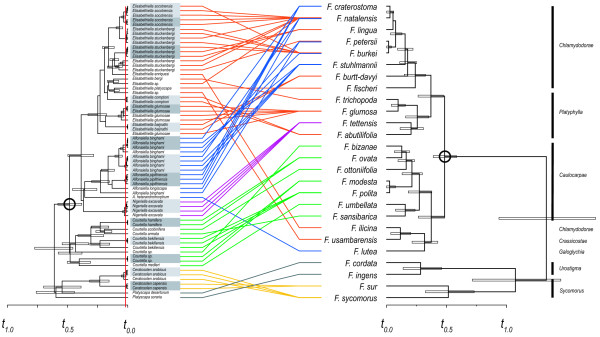
**Tanglegram of pollinator lineages and host *Ficus *species**. Strict molecular clock (*COI*) fig wasp and (*ETS *&* ITS*) *Ficus *ultrametric phylogenies were generated using BEAST. Coloured lines connect wasp specimens with the fig species they were collected from (each connecting line is coloured according to fig wasp genus). A relative time scale is given. The nodes used to calibrate the divergence timing estimates are indicated by large open circles. Horizontal open bars represent the 95% upper and lower posterior density intervals around the mean node age. Black vertical bars on the right indicate *Ficus *subsections. The red vertical line indicates the threshold at which the Yule pure-birth model is inferred to have switched to a neutral coalescent model. Grey boxes indicate clades falling within the coalescent region.

The GMYC likelihood test was robust to both molecular clock and relaxed clock assumptions and identified 40 'entities' or clades from a total of 65 terminal taxa, which are inferred to include coalescing lineages below the level of species (Figure 1). A total of 16 clusters represent clades that comprise potential below-species divergence at the *COI *locus. The inferred shift at which species diversification changes to within-lineage processes was significant (*P *= 0.000). The threshold time (-0.3040073) was converted to a relative time scale. The likelihood of the null model was less than the alternative model (*Ln *null model = 79.27529; *Ln *GMYC model = 96.37995) indicating a switch between-species branching according to a Yule pure-birth model and neutral coalescence. Within 5 of these 'coalescing' clades are host associations with more than one *Ficus *species. *Elisabethiella stuckenbergi *(Grandi), *E. glumosae *Wiebes, *Alfonsiella Binghami *Wiebes, and *Courtella bekiliensis *(Wiebes) show species level divergences within each of these 'species groups': (i) *E. socotrensis *(Mayr) was collected from *F. natalensis natalensis *Hochst. and *F. burkei *(Miq.) Miq.; (ii) *E. stuckenbergi *(between 3 clades indicating coalescence) from *F. burkei, F. natalensis*, and *F. lingua lingua *De Wild. & T. Durand, *F. petersii *Warb.; and (iii) *A. binghami *(between 2 clades showing coalescence, and a species-level lineage) from *F. craterostoma *Mildbr. & Burret, *F. stuhlmannii *Warb., *F. petersii*, and *F. natalensis*. The log-lineages through time plot (Additional file 3) shows a sharp increase in lineage accumulation that represents the inferred shift between speciation and coalescent processes.

### Statistical parsimony and AMOVA

The statistical parsimony analysis partitioned the *COI *sequence data into a series of 14 haplotype networks possessing two or more samples and 20 singleton haplotypes (Additional file 4). Of the Agaonidae pollinator networks, at least 4 groups (species) were collected from more than 2 *Ficus *species. Differentiation among species was significant (*P *< 0.000) at *F_ST _*= 0.78. Analysis of molecular variance showed significant levels of differentiation for all species (a 'species' compared to all other 'species' groups) except for within the *A. binghami *group (group specific *F_ST _*= 0.55) that was much lower than the those of other groups that ranged from *F_ST _*= 0.77 to 0.86 (Table 1). Species-specific fixation indices ranged from *F_ST _*= 0.55 to *F_ST _*= 0.86 (Table 2) and were largely in the vicinity of the latter. Both GMYC and statistical parsimony tests divide *E. stuckenbergi *into at least three putative species clades with substantial differentiation between each. Statistical parsimony tended to be more sensitive to differentiation among putative species lineages within *A. binghami *and *E. socotrensis*. Species delimitation that relies on single loci is not conclusive and has to be treated with caution. However, divergence at the *COI *locus provides credible evidence of negligible differentiation among pollinator lineages sampled from different *Ficus *species and is important to the interpretation of host species associations and co-diversification.

**Table 1:** AMOVA tests of between lineage differentiation

	**d.f**.	Sum of squares	Variance components	Percentage variation
Among	13	973.921	22.74287	78.47
Within	29	180.986	6.24089	21.53
Total	42	1154.907	28.98375	
***F_ST_*:**	**0.78468**	***P *< 0.000**		

Fixation indices (*F_ST_*) among each fig wasp species at the *COI *locus.

**Table 2:** AMOVA tests of species-specific differentiation

Group #	Species	*F_ST_*
1	*A. binghami*	0.54792
2	*A. pipithiensis*	0.83716
3	*E. socotrensis*	0.81126
4	*E. stuckenbergi 1*	0.84645
5	*E. stuckenbergi 2*	0.85222
6	*E. stuckenbergi 3*	**0.81363**
7	*E. comptoni*	0.85175
8	*E. glumosae*	0.85598
9	Unknown sp.	0.85175
10	*C. hamifera*	0.85175
11	*C. bekiliensis*	0.85598
12	*N. excavata*	0.80622
13	*C. arabicus*	0.77128
14	*C. capensis*	0.84751

Species-specific fixation indices (*F_ST_*) at the *COI *locus.

### Phylogenetic congruence analyses

A comparison between 42 terminal pollinator taxa with 26 *Ficus *species resulted in non-significant congruence using ParaFit and under particular event cost schemes in TreeFitter tests. The application ParaFit tests a global null hypothesis that the association between the 'host' and 'parasite' phylogenies is independent. The global test of independence was insignificant (*P *= 0.18). Tests on individual associations indicate that the inferred lack of cophylogeny was a result of only 4 of the 42 links being significant (*P *< 0.05) in their contribution to the global test statistic. Instances of host switching relative to codivergence events were not particularly sensitive to cost assignment modulation and indicated preponderance to more switching than codivergence (Table 3). When event costs were set to default values (0,0,1,2) or where switching was similarly down-weighted (0,1,1,1), instances of codivergence (13-14; *P *< 0.05 and 9-13; *P *< 0.05 respectively) by comparison to host-switching (20-23; *P *< 0.05 and 23-29; *P *< 0.05) were significantly less (*P *< 0.001) where the total cost of random trees was greater than observed associations. These results are perhaps realistic given undisputed species-level phylogenetic congruence shown in the literature (i.e. pollinator fig wasp host-switching is less likely than codivergence). Significant instances of duplication events of pollinator lineages (5-7; *P *< 0.05) were indicated by the analysis using default cost assignments.

**Table 3:** Event-based tree reconciliation

Event costs	Cost	Codivergence	Duplication	Sorting	Switching
0,0,1,2***	50	13-14*	5-7**	4-10	20-23**
1,1,1,1	41	0-9*	3-9	0-0	27-38**
0,1,1,1***	32	9-13*	3-5	0-4	23-29**
1,0,1,1	32	0-5*	9-9	0-0	27-32**
1,1,0,1	41	0-18	3-41	0-271	0-38
1,1,1,0	3	0-0	3-3	0-0	38-38

Event-based analysis of host associations between fig wasp lineages and *Ficus *species implemented in TreeFitter. Event costs weighting schemes (left column) are ordered as follows: codivergence, duplication, sorting, and switching. Redundant exemplars of the same fig wasp and host-species association were removed for the purposes of the test. * Observed event significantly more than randomised trees *P *< 0.05. ** Observed events significantly less than randomised trees *P *< 0.05. *** Total event costs of observed trees were significantly less than the randomised trees *P *< 0.05.

The general agreement between reconciliation tests of alternative topologies (not shown) indicates switching events exert a considerable influence, but does not alter the approximate numbers co-divergence events. Phylogenetic uncertainty inherent in these types of analyses, acts to obscure accuracy in tree topology and divergence estimates. The results show that the tests of congruence are robust to changes in topology. Incomplete representation of *Ficus *population genetic variation might bias the frequency of codivergence events. However, the results provide a reasonable indication of switching and duplication in terms of *Ficus *species. Overall, these results show that pollinators are highly constrained to *Ficus *subsections and switching between these higher taxonomic scales is rare and likely represents very ancient events (Figure 1). For instance, host associations of *Allotriozoon heterandromorphum *Grandi and *F. lutea *Vahl, *E. enriquesi *(Grandi) and *F. ilicina*, and an undescribed *Elisabethiella *species and *F. usambarensis *Warb. Divergence timing estimates of both pollinator and *Ficus *phylogenies show a high incidence of overlap among the 95% upper and lower posterior density intervals around the mean node ages. Regardless of this uncertainty, host-switching events of single pollinator species appears to occur between *Ficus *lineages that share a common ancestor that predates the origin of the pollinator populations. The age of the split between subsections *Sycomorus/Urostigma *and the subsections of section *Galoglychia *is inferred as relatively ancient. Inconsistency between the performance of *COI *and *ETS/ITS *fragments and sampling effects likely explains this discrepancy in the reconstruction of deep nodes.

## Discussion

Species of *Elisabethiella *pollinating fig wasps reproduce on at least three *Ficus *subsections. Long branches tend to be associated with the most extreme instances of phylogenetic incongruence implying ancient host shifts have shaped contemporary relationships. Although reconciliation tests indicated significant phylogenetic correspondence, host-switching patterns among pollinator lineages inferred to be below species level were more prolific than codivergence patterns. The discordance between the pollinator and *Ficus *phylogenies was in part due to restricted transfer of *Elisabethiella *and *Alfonsiella *populations amongst closely related host species in subsection *Chlamydodorae*. Reconciliation analyses also detected duplication patterns that can be partly explained by fig wasp differentiation via host switching, or in allopatry on the same host species, followed by secondary contact with sibling lineages. Repeated evidence of divergent putative species-lineages within what is considered the same 'good morphological species' of pollinator is consistent with a time lag between speciation of pollinators and that of hosts. Together these findings suggest that diversification in pollinating fig wasps is driven by preferential host-use amongst closely related species in conjunction with ecological factors contributing to speciation.

**Figure 2:**
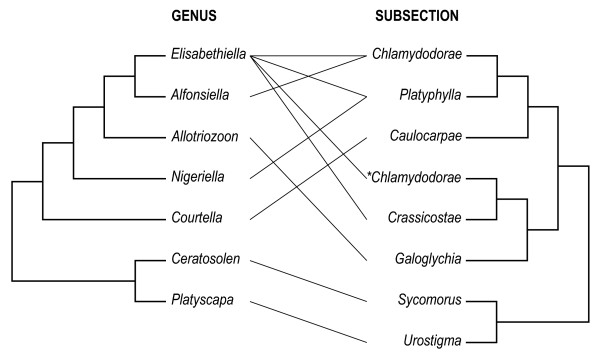
**Higher level phylogenetic congruence**. Broader taxonomic scale depiction of pruned phylogenies showing host associations between fig wasp genera and *Ficus *subsections. Note that the genus *Elisabethiella *has a wider host subsection range than the other genera sampled. Host switching has occurred between relatively divergent clades that comprises at least two different subsections. *Requires revision or poorly resolved.

Phylogenetic reconciliation analyses (Table 3) show significant evidence of independence between *Ficus *and pollinator divergence due to either 'duplication' or 'host switching.' Both these types of patterns largely preclude synchronistic wasp-host diversification hypotheses. However, the phylogenetic discordance reported here might also be a function of the dissimilar taxonomic scales used to test associations between wasps and hosts. For instance, breakdown in phylogenetic concordance patterns has been argued to be partly the result of genetic introgression and hybridization across different fig species [8,38,40]. The *Ficus *phylogeny (Additional file 2) considered only species and not intraspecific variation. It is difficult to determine the distribution of host traits that are actually targeted by pollinators and how closely correlated these traits are to host phylogeny. Pollinator transfer between different *Ficus *species should increase the tendency for post-speciation introgression between host lineages [9] and presumably influence the evolution of fig wasp and host fig phenotypes. It would appear by the results presented in this study that the intensity of this type of genetic exchange should be predomiantly confined among closely related host species. Therefore, underestimation of intraspecific genetic variation of hosts might result in overestimation of phylogenetic incongruence at the species level. However, multiple host species associations with a single pollinator species and associations of *Elisabethiella *with several host subsections (Figure 2) indicates horizontal transfer between different *Ficus *species does play some role in the evolution of wasp and host fig phenotypes.

Generally, host-plant switches can occur between distantly [41-45] or more closely related host lineages [20,46,47]. Arguably, pollinator fig wasps occupy the most extreme end of host specificity observed in insect-plant associations. Horizontal transfer by pollinator wasps between host lineages appears to be the most important contributor to the phylogenetic incongruence shown by this study (Table 3). The patterns of incongruence can be interpreted in a number of ways. The empirical evidence of 'host switching' derived from the reconciliation simulations does not discriminate between the strength of fidelity between natal and novel host species or the duration of associations with different host lineages. Thus, intensity of pollinator specialisation for a given host species and the magnitude of gene flow between pollinator populations sharing different host species are difficult to decipher [17,48]. The *COI *haplotype tree shows *E. socotrensis, E. stuckenbergi*, and *A. binghami *all possess low-level (below species) genetic divergence among lineages on different host species (Figure 1). *Alfonsiella binghami *populations are able to reproduce on *F. stuhlmannii, F. natalensis, F. burkei, F. petersii*, and *F. craterostoma *(Figure 1). However, this does not imply reproductive isolation among populations on different the host species. It is uncertain whether the pollinator populations that use alternative host species for reproduction are evolutionarily viable and become good species rather than go extinct. Conversely, *Elisabethiella glumosae, E. socotrensis, E. stuckenbergi*, and *A. binghami *show considerable divergence at the *COI *locus between lineages on the same host species. As these incipient/putative species of *Elisabethiella *and *Alfonsiella *belong to lineages that are mostly monophyletic and not polyphyletic, the haplotype tree (Figure 1) likely shows orthologous alleles that have evolved from a common ancestor [49]. One exception was the paraphyletic relationship *Elisabethiella glumsosae *has with another lineage of this 'morphological species' and might reflect phylogenetic uncertainty or a morphologically similar but different species.

Together, phylogenetic inference, statistical parsimony, and reconciliation analyses strongly imply that horizontal transfer among host species and lineage duplication are responsible for much of the phylogenetic incongruence. Previous work [8,13] has shown evidence of both lineage duplication and host switching while more recent studies have debated the role extinction and duplication play in explaining phylogenetic incongruence [30,38]. The results presented in this study do not refute any of these arguments, especially as extinction scenarios are difficult to substantiate. Assuming wasp and host fig extinction was negligible, more pollinator species should have evolved in relation to host species given duplication or host switching, and this is consistent with other studies [8,13,31]. Pollinator lineage 'duplication' was the least frequent of the significant tree reconciliation scenarios (Table 1). Eventual extinction by competitive exclusion of one pollinator species that shares a host with another has been used to explain phylogenetic incongruence [30]. Phylogenetic relatedness has been shown to be a good predictor of competitive exclusion [50]. However, this hypothesis relies on saturation of available resources or niche and does not seem applicable to *Ficus*. Resource utilisation of ephemeral and unpredictable host species by fig wasps in general, occurs at low levels [51]. Pollinator recruitment does not occur for all available *Ficus *at any given period and should relax selection based on competitive exclusion. Duplication is characteristic of insect populations being isolated from one another while the host is not and does not imply that a duplicated lineage has to arise on the same host species nor in allopatry [52]. Host switching among different *Ficus *species in sympatry might be responsible for pollinator lineage duplication via secondary colonisation of an ancestral host species that still supports a closely related pollinator lineage [53]. Differentiation in allopatry between populations supported by the same host species followed by secondary contact would result in the same incongruence pattern. Presumably, duplication results from either transfers among closely related sympatric host species, or in allopatry on the same host species, or a combination of both. The contribution of any one of these processes should depend on limitations set by ecological, physiological, and morphological specialisation [54,55].

It is difficult to speculate accurately about the contribution of extinction to the observed phylogenetic incongruence shown in this or any other study. The relatively inconsequential number and non-significance of sorting events (pollinator extinction independent of the host) detected in this study (Table 3) might be a consequence of low extinction probability overall [56]. Local extinctions have been caused by drought where up to 17 pollinator species became locally extinct [57]. This could ultimately lead to the local extinction of the corresponding figure. However, co-extinction of pollinator species might not be as influential when host species are broadly distributed over large regions such as Southern Africa. Local extinction of pollinators might not be a threat due to relatively long-lived fig species and re-colonization from other source populations [58]. The relative lack of pollinator lineage loss from host lineages might be disguised by a predominance of co-extinction, but would not be detected without fossil evidence [59-61]. Low levels of local endemnicity of *Ficus *hosts sampled by this work suggest extinction might not play a substantial role in the low levels of phylogenetic correspondence.

Evidence of phylogenetic congruence is indicative of restricted switching among closely related hosts. Host species of *Elisabethiella *and *Alfonsiella *species are largely sympatric over their host distributions [35]. Host switching *per se *could lead to sympatric speciation via disruptive selection. For sympatric speciation to operate, selection must overcome gene flow where different host species are geographically arranged such that insect dispersal does not limit migration between them [62]. Fig wasp dispersing capability is believed to be substantial [63]. Trade-offs between alternative host-species that reduce fitness on one host and increase fitness on another is necessary for disruptive selection and sympatric speciation [64]. However, the best-known example of disruptive selection is inconclusive and might have involved divergence in allopatry preceding secondary contact [6,65,66]. Furthermore, it is difficult to test hypotheses that reject allopatry [67]. Selection imposed by ecological interactions might also induce speciation. Host adaptation to different habitat types in close proximity suggests ecologically driven speciation [68]. Habitat selection and physiological tolerance limitations of pollinator populations to different environmental variables [55] might support ecologically-driven speciation [69] in both pollinator and *Ficus *lineages. Host transfers among closely related *Ficus *species should be linked to life history constraints or preferential targeting of traits possessed by closely related phenotypes. Fig wasp host-use behaviour has been shown to tightly adhere to specific volatile cues emitted by their host species [70-72]. This suggests that fig wasps track plant secondary chemistry where colonisation of a novel host occurs between chemically similar lineages, assuming convergence of chemical traits is absent [73]. Such behaviour is characterized by delayed associate speciation and has been described as host tracking [74,75]. Host tracking of gall wasps that specialise on oaks [76] appears to be at least partially conserved due to galling life history constraints. Rare and periodic switches were shown to occur between more distantly related host section lineages and this is similar to the patterns of host-use by pollinating fig wasps. Limitations set by galling behaviour of pollinating fig wasps might have consequences for the breadth of host phenotypes that can be used for reproduction and between which pollen is transferred.

## Conclusions

Significant levels of concordant and independent divergence between pollinating fig wasp lineages and *Ficus *species revealed several processes important to the evolution of the mutualism. This study supports recurrent backcrossing between closely related *Ficus *species and very rarely between distantly related host lineages. Evidence showing pollinator lineage duplication could have arisen both in sypmatry by switching among different host species, or allopatrically on the same host species, followed by secondary contact between sibling lineages. Unrealized within-species genetic variation of both fig wasps and *Ficus *need to be accounted for to accurately identify causal links between host lineage conservatism and speciation. Analyses using single pollinator species-representatives might result in misleading interpretation of host associations and obscure patterns relevant to speciation processes. This work shows that the propensity for host transfer among closely related *Ficus *species is variably independent of host speciation and permits ecological infuences on the speciation of pollinating fig wasps.

## Methods

### Sampling

Most African *Ficus *are recognised in section *Galoglychia *[77,78] with fewer in sections *Sycomorus *and *Urostigma *that are most strongly represented in the Indo-Australasian region [79]. A large majority of African *Ficus *are monoecious, containing male and female flowers within the same syconium. Multiple specimens of pollinating fig wasp species (Agaonidae) from the genera *Elisabethiella *Grandi, *Alfonsiella *Waterston, and *Courtella *Kieffer were most abundant in our collections and served as the focal group in this investigation. All known available and compatible sequence data was incorporated into the study. No DNA material was available for the genera *Blastophaga *and *Kradibia *that each comprises single species. Other less well-represented taxa were used in the analysis to improve species delimitation tests and provide a broader taxonomic context. These comprised species from the genera *Allotriozoon *Grandi, *Nigeriella *Wiebes, *Ceratosolen *Mayr, and *Platyscapa *Motschoulsky. Together, the sample included 24 of the 71 described Afrotropical agaonid species. Both pollinator and *Ficus *outgroup constraints were based on previous inferences [22]; however, taxonomic distinctions among *Ficus *[9,38] and pollinator [37] species remain controversial. All pollinator sequences (Additional file 5) were sequenced *de novo *from recent tissue collections with voucher specimens deposited at the Iziko South African Museum. *Ficus *sequences were sourced from GenBank ([22,80]; Additional file 6).

### Phylogenetic inference

Phylogenetic inferences of pollinator and *Ficus *sequence data were used for relative divergence timing estimation and for tests of congruence between them. To generate phylogenetic inferences among the wasps, fragments of mtDNA cytochrome oxidase one (*COI *~ 620 bp), cytochrome b (*Cytb *~ 380 bp), and nuclear DNA (nDNA) elongation factor-one alpha F2 copy (*EF-1α *~ 500 bp) were sequenced. A comprehensive explanation of DNA extraction, PCR, and alignment protocols is given in [81]. The species delimitation test requires an (mtDNA) ultrametric tree. Substitution rates of *COI *mtDNA tend to be higher than in nuclear protein coding genes and therefore more prone to saturation bias that impedes deep node resolution. In order to implement reasonable prior tree topology constraints for ultrametric pollinator tree inference and for co-phylogenetic analyses, we used species for which multiple gene fragments including nDNA were available to infer a phylogeny using Bayesian and parsimony approaches. Sequence data of up to 767 bp's of a ribosomal internal transcribed spacer (*ITS*) and up to 479 bp of external transcribed spacer (*ETS*) were used to infer *Ficus *species phylogenies under Bayesian and parsimony methods. Analyses presented in this work assume the *Ficus *species phylogeny is fully resolved and does not consider population-level genetic variation influence on host associations. Species-level appraisal of host lineages does provide evidence of comparative genetic distances for instances of departures from one-to-one species specificity. Sequences of *ITS *and *ETS *were amplified using the protocol outlined in previous work [80].

A Bayesian approach implemented in MrBayes 3.1.1 [82] was used to partition the *COI, Cytb*, and *EF-1α *data into gene fragments and also codon positions. The *Ficus *sequence data was partitioned into *ITS *and *ETS *for the Bayesian phylogenetic analyses. A general time reversible DNA substitution model was used with gamma distributed (+G) rates with a proportion of invariant sites (+I). Posterior probabilities and mean branch lengths were derived from 15000 post-burnin trees sampled every 1000 trees from generations 5 to 20 million. Four separate Bayesian reconstructions were run to verify consistency of phylogenetic outcomes. The consensus trees were derived from post-burnin generations of Markov chains that had reached apparent stationarity. Convergence was assessed using the MCMC Tracer Analysis Tool v.1.4.1 [83] by plotting the log likelihoods to assess the point in the chain where stable values were reached and with the standard deviation of split frequencies of all runs. Parsimony bootstrap analysis implemented using PAUP version 4.0b10 [84] was used to assess the robustness of the Bayesian consensus phylogeny. The parsimony bootstrap consensus trees were derived from a search consisting of 500 bootstrap replicates using a full heuristic search. To calculate bootstrap support, we used branch-swapping by stepwise addition on best trees only, 100 random additional sequences holding 10 trees at each step, and the TBR search algorithm.

### Within-lineage diversification

To distinguish population-level processes from species diversification we used the generalised mixed Yule coalescent (GMYC) likelihood test [85]. The mixed Yule coalescent analysis approach has been shown to be relatively robust to sampling effects, marker use, the numbers of markers used [86]. The test uses models that describe ultrametric phylogenetic trees that have either within-population coalescent branching or species branching signatures. The GMYC test assumes between-species branching according to a Yule pure-birth model [87] and within-species neutral coalescent model [88]. A likelihood test of the mixed model estimates the shift from speciation to within-population branching. Although the threshold at which this split occurs might not reflect true diversification processes across all lineages in large trees [89], the approach has been used with high rates of success on preliminary species delimitation of diverse groups including insects [85]. The test is intended to be complementary to traditional morpho-taxonomic approaches, but useful for detecting the presence of within-species diversification. The GMYC test was implemented using the 'R' [90] package SPLITS (available from: http://R-Forge.R-project.org). An ultrametric tree reconstruction was generated using a strict molecular clock (the GMYC test is based on the strict clock assumption) implemented in BEAST v.1.4.8 [91] with gamma distributed invariant sites, GTR substitution prior, empirically estimated base pair frequencies, and unlinked 1^st ^plus 2^nd ^positions, and 3^rd ^codon position. We also used an ultrametric tree based on a relaxed clock using the same priors as above. To compare branching time estimates between fig wasps and figs, we reconstructed a molecular clock *Ficus *tree under the same priors, but with linked codon positions. We set topology priors that constrained the clade of each fig wasp genera and the monophyletic subsections as inferred by our Bayesian consensus. Sequence data comprising approximately 680 bp's of a *COI *mtDNA fragment was used in GMYC tests of the fig wasps and 1345 bp's of the ITS and ETS regions for the *Ficus *analysis. All available sequence data for *Elisabethiella *and *Alfonsiella *were used in the analysis as GMYC test performance is optimised using larger sample numbers comprising non-zero branch length information [92]. We generated a log-lineages through time plot to visualize the 'coalescent region' or shift between species and population branching patterns. Outgroup constraints were the same as for the phylogenetic inferences above. We also constrained the topology of fig wasp genera in the BEAST analysis and verified the generic relationships with the abridged multiple gene fragment phylogenetic inference. A tanglegram between the two BEAST molecular clock inferences was constructed by hand to illustrate fig wasp associations with *Ficus *species.

### Statistical parsimony and AMOVA

Statistical parsimony implemented using TCS v. 1.8 [93] was used to partition the *COI *sequence data into independent networks connected by non-homoplasious mutations. Statistical parsimony estimates the maximum number of single substitutions among haplotypes (referred to as the connection limit) preceded by the connection of haplotypes into a network differing by increasing numbers of single site changes [94]. All taxa within a given genus were analysed using statistical parsimony and the resulting networks assumed to be putative genetic species [95,96] for AMOVA below. A 95% connection limit probability was used, where gaps were treated as missing with no connection limit step priors. *F_ST _*coefficients (at the 0.05 significance level) were estimated using AMOVA as implemented in Arlequin v. 3.1. [97] by permutating groups (species) of haplotypes among networks to assess fixation indices among the putative species. The *F_ST _*coefficient is the proportion of the total genetic variance within a subpopulation (*S*) relative to the total genetic variance (*T*) and ranges from 0 to 1. A high *F_ST _*implies substantial differentiation among groups and was expected under the hypothesis of statistical parsimony networks representing putative species. The *P*-value of each test is the proportion of permutations resulting in an *F_ST _*value larger or equal to the observed proportions. We used 10,000 permutations to estimate *F_ST _*and *P-*values and a gamma distribution of 0.5 that was determined using Modeltest v.3.0 [98].

### Codiversification analyses

A statistical test of congruence implemented in ParaFit [99] and a tree reconciling method implemented in TreeFitter [100] were used to investigate the magnitude of phylogenetic correspondence between the fig wasp *COI *haplotype tree and the *Ficus *species tree. These methods were selected on the basis of being able to accommodate the unequal numbers of terminal taxa between host and parasite tree, and to gauge both a global indication of congruence and the relative contribution of host switching (parasite horizontal transfer to another host lineage), duplication (parasite speciation without host speciation), sorting (parasite loss from host lineage) and codivergence (co-cladogenesis of parasite and host) patterns to tree correspondence. Both methods assume that the trees are fully resolved without polytomies. ParaFit was used to test a global null hypothesis of independent host-parasite associations revealed by two phylogenetic trees. This approach is not directed at reconstructing a putative history among hosts and parasites. The associations between the phylogenies are randomized and tested. Patristic distance matrices were calculated for both fig wasp (*COI*) and *Ficus *(*ETS *and *ITS*) sequence data using PAUP and then converted to principal coordinates using the R package ade-4 [101]. A matrix explaining the associations between hosts and parasites is permutated at random and used in conjunction with the other two matrices to produce a test statistic. We used 9999 permutations to calculate the test statistic. By comparison, TreeFitter is an event-based parsimony method that is useful for exploring the relationships between gene trees (parasite tree) and species trees (host tree). In the absence of reliable species delimitation, Ricklefs and colleagues [102] treated each haplotype as a separate entity. Inclusion of 'associate' lineages below species (e.g. entities) can result in reflecting excessive duplication (speciation within host lineages). In order to minimize this effect, all sequence data of replicate host-pollinator associations were removed for both congruence analyses. The number of codiversification, sorting, duplicating, and switching events was tested against random datasets drawn by a heuristic search of tree space generated by the Markov process. TreeFitter calculates the event frequencies according to the minimum cost solutions for various runs where the host tree was permutated. Default cost-assignments were used (co-divergence equal to 0, duplication equal to 0, lineage sorting set at 1, and switches a cost of 2; i.e. 0,0,1,2) in addition to separate analyses involving equal and down weighting of costs of each event in different tests (1,1,1,1; 0,1,1,1; 1,0,1,1; 1,1,0,1; 1,1,1,0).

## Authors' contributions

The conception and design of the analysis was developed by MJM. Bench work, sequence alignments, data analyses, interpretation of results, and manuscript drafting were conducted by MJM. Morphological appraisal of specimens, intellectual contributions, and interpretation of analyses were given by SvN. Field collections were contributed to by SvN and MJM. All authors have read and approved the final manuscript.

## Supplementary Material

Additional file 1 **Bayesian (A) and parsimony bootstrap (B) consensus fig wasp phylogenies generated from *EF-1α, COI*, and *Cytb *sequence data**. Click here for file

Additional file 2 **Bayesian (A) and parsimony bootstrap (B) consensus *Ficus *species phylogenies generated from *ETS *and *ITS *sequence data**. Click here for file

Additional file 3 **A log-lineages through time plot derived from the *COI *marker with grey vertical line indicating the threshold at where species diversification shifts to coalescent processes**. Click here for file

Additional file 4 **Groups determined by statistical parsimony and GMYC tests for population-level entities where there was more than one in the group**. Click here for file

Additional file 5 **Collection details of fig wasp specimens**. Click here for file

Additional file 6 **GenBank Accession Numbers *Ficus *species**. Click here for file
