# Supplementary material for: Codivergence and multiple host species use by fig wasp populations of the Ficus pollination mutualism
Source: BMC Evol Biol. 2012 Jan 3;12:1. doi: 10.1186/1471-2148-12-1 (PMC3299616; doi:10.1186/1471-2148-12-1)
Supplement: Additional file 1 — Bayesian (A) and parsimony bootstrap (B) consensus fig wasp phylogenies generated from EF-1α, COI, and Cytb sequence data. [file 1471-2148-12-1-S1.PDF]

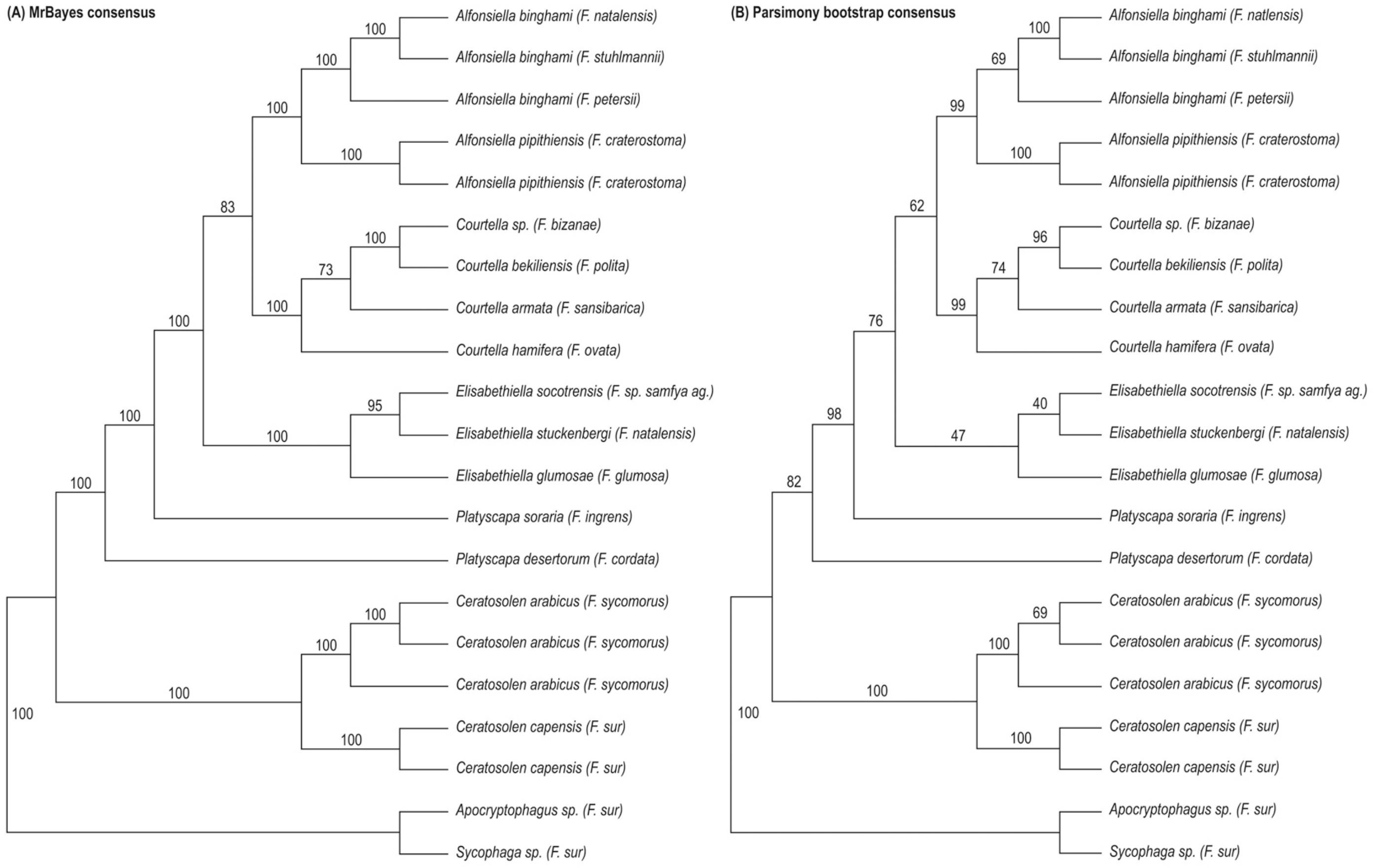


Additional file 1: Bayesian (A) and parsimony bootstrap (B) consensus pollinator fig wasp phylogenies generated from *EF-1α*, *COI*, and *Cytb* sequence data.
