# Supplementary material for: Codivergence and multiple host species use by fig wasp populations of the Ficus pollination mutualism
Source: BMC Evol Biol. 2012 Jan 3;12:1. doi: 10.1186/1471-2148-12-1 (PMC3299616; doi:10.1186/1471-2148-12-1)
Supplement: Additional file 2 — Bayesian (A) and parsimony bootstrap (B) consensus Ficus species phylogenies generated from ETS and ITS sequence data. [file 1471-2148-12-1-S2.PDF]

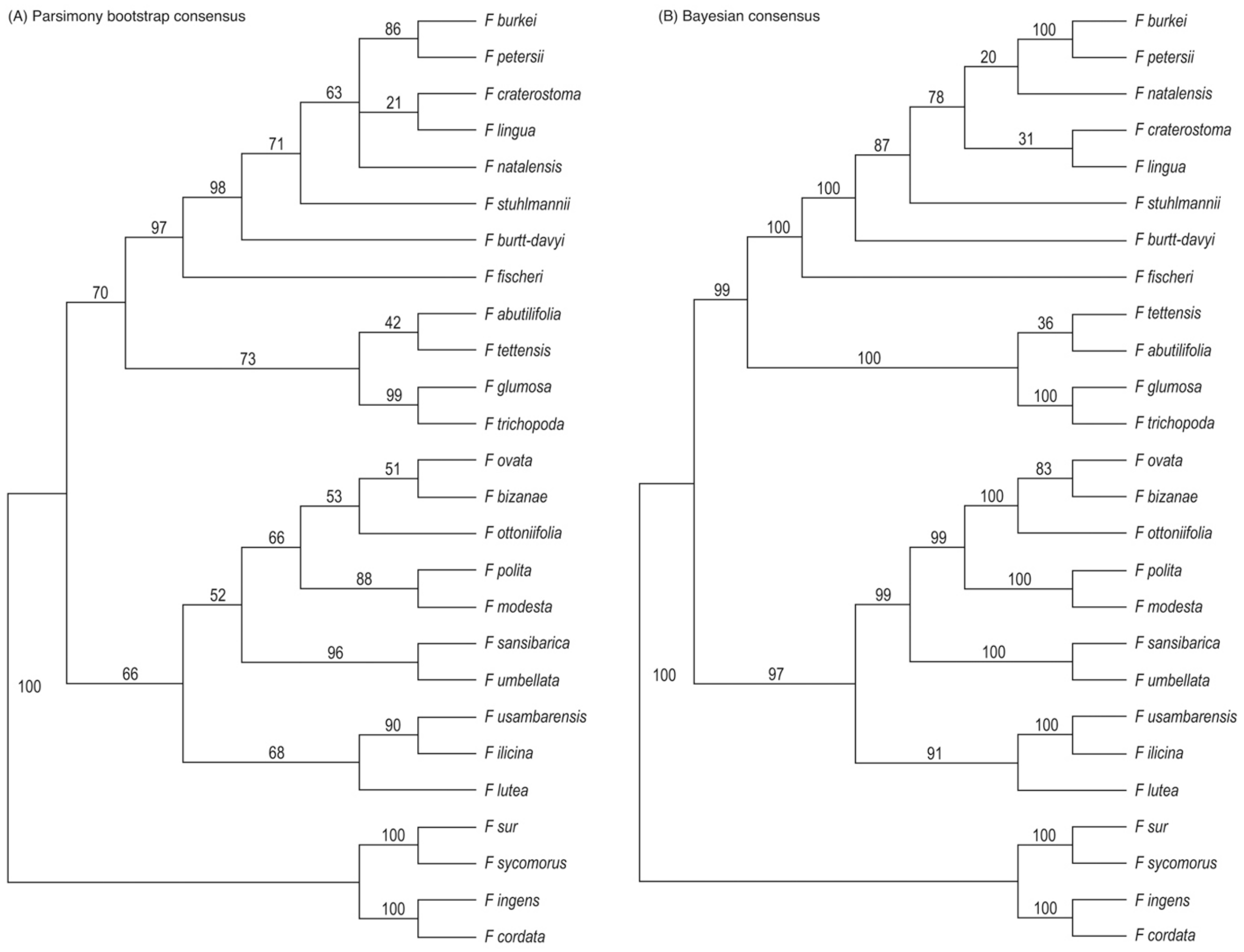

Additional file 2: Bayesian (A) and parsimony bootstrap (B) consensus *Ficus* species phylogenies generated from *ETS* and *ITS* sequence data.
