# Supplementary material for: Codivergence and multiple host species use by fig wasp populations of the Ficus pollination mutualism
Source: BMC Evol Biol. 2012 Jan 3;12:1. doi: 10.1186/1471-2148-12-1 (PMC3299616; doi:10.1186/1471-2148-12-1)
Supplement: Additional file 3 — A log-lineages through time plot derived from the COI marker with grey vertical line indicating the threshold at where species diversification shifts to coalescent processes. [file 1471-2148-12-1-S3.PDF]

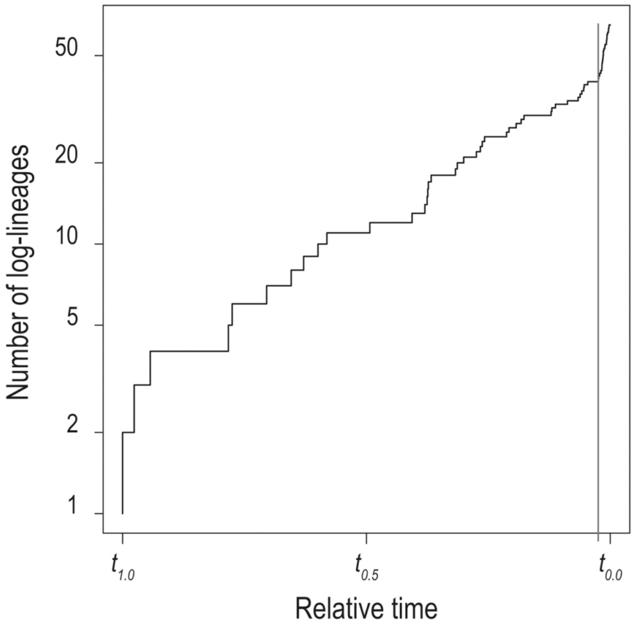

Additional file 3: A log-lineages through time plot derived from the *COI* marker with grey vertical line
