# Supplementary material for: Codivergence and multiple host species use by fig wasp populations of the Ficus pollination mutualism
Source: BMC Evol Biol. 2012 Jan 3;12:1. doi: 10.1186/1471-2148-12-1 (PMC3299616; doi:10.1186/1471-2148-12-1)
Supplement: Additional file 4 — Groups determined by statistical parsimony and GMYC tests for population-level entities where there was more than one in the group. [file 1471-2148-12-1-S4.PDF]

Additional file 4: Groups determined by statistical parsimony and GMYC tests for population-level entities for cases where there was more than one in the group.

**Species**	***Ficus* host**	***Ficus* sub-section**	**Collection/ Accession**	**Stat. Pars. Network**	**GMYC Cluster**
A. binghami	*F. burkei*	*Chlamydodorae*	AY014974	SP A	GMYC A
A. binghami	*F. stuhlmannii*	*Platyphyllae*	AJ971648	SP A	GMYC A
A. binghami	*F. stuhlmannii*	*Platyphyllae*	MW06-F60	SP A	GMYC B
A. binghami	*F. stuhlmannii*	*Platyphyllae*	SA05-F55B	SP A	GMYC B
A. binghami	*F. natalensis*	*Chlamydodorae*	MW06-F89	SP A	GMYC B
A. binghami	*F. stuhlmannii*	*Platyphyllae*	KN08-F64	SP A	GMYC B
A. binghami	*F. petersii*	*Chlamydodorae*	SA05-F45	SP A	GMYC C
A. pipithiensis	*F. craterostoma*	*Chlamydodorae*	AJ971649	SP B	GMYC D
A. pipithiensis	*F. craterostoma*	*Chlamydodorae*	KN08-F15	SP B	GMYC D
A. pipithiensis	*F. craterostoma*	*Chlamydodorae*	KN08-F52	SP B	GMYC D
E. socotrensis	*F. burkei*	*Chlamydodorae*	AM260705	SP C	GMYC E
E. socotrensis	*F. natalensis*	*Chlamydodorae*	AM260706	SP C	GMYC E
E. socotrensis	*F. natalensis*	*Chlamydodorae*	AM260707	SP C	GMYC E
E. socotrensis	*F. natalensis*	*Chlamydodorae*	SA05-F08	SP C	GMYC F
E. socotrensis	*F. natalensis*	*Chlamydodorae*	SA05-F08	SP C	GMYC F
E. stuckenbergi	*F. natalensis*	*Chlamydodorae*	AJ971651	SP D	GMYC G
E. stuckenbergi	*F. burkei*	*Chlamydodorae*	SA06-F98	SP D	GMYC G
E. stuckenbergi	*F. burkei*	*Chlamydodorae*	SA05-F28	SP D	GMYC G
E. stuckenbergi	*F. burkei*	*Chlamydodorae*	KN08-F68	SP D	GMYC G
E. stuckenbergi	*F. burkei*	*Chlamydodorae*	AM260704	SP E	GMYC H
E. stuckenbergi	*F. lingua*	*Chlamydodorae*	MW06-F86	SP E	GMYC H
E. stuckenbergi	*F. lingua*	*Chlamydodorae*	MW06-F88	SP E	GMYC H
E. stuckenbergi	*F. natalensis*	*Chlamydodorae*	SA05-F08	SP F	GMYC I
E. stuckenbergi	*F. burkei/natalensis*	*Chlamydodorae*	ZA06-F14	SP F	GMYC I
E. comptoni	*F. abutilifolia*	*Platyphyllae*	AJ971652	SP G	GMYC J
E. comptoni	*F. abutilifolia*	*Platyphyllae*	SA05-F23	SP G	GMYC J
E. glumosae	*F. glumosa*	*Platyphyllae*	SA05-F19	SP H	GMYC K
E. glumosae	*F. glumosa*	*Platyphyllae*	SA06-F97	SP H	GMYC K
Courtella sp.	*F. modesta*	*Caulocarpae*	MW06-F70	SP I	GMYC L
Courtella sp.	*F. modesta*	*Caulocarpae*	MW06-F69	SP I	GMYC L
C. bekiliensis	*F. polita*	*Caulocarpae*	AY014977	SP J	GMYC M
C. bekiliensis	*F. polita*	*Caulocarpae*	SA06-F95	SP J	GMYC M
C. hamifera	*F. ovata*	*Caulocarpae*	ZA06-F17	SP K	GMYC N
C. hamifera	*F. ovata*	*Caulocarpae*	ZA06-F19	SP K	GMYC N
N. excavata	*F. tettensis*	*Platyphyllae*	SA05-F04	SP L	GMYC O
N. excavata	*F. tettensis*	*Platyphyllae*	SA05-F04	SP L	GMYC O

N. excavata	*F. tettensis*	*Platyphyllae*	AJ971655	SP L	GMYC O
C. arabicus	*F. sycomorus*	*Sycomorus*	KN08-F56	SP M	GMYC P
C. arabicus	*F. sycomorus*	*Sycomorus*	KN08-F58	SP M	GMYC P
C. arabicus	*F. sycomorus*	*Sycomorus*	KN08-F62	SP M	GMYC P
C. capensis	*F. sur*	*Sycomorus*	SA05-F27	SP N	GMYC Q
C. capensis	*F. sur*	*Sycomorus*	KN08-F01	SP N	GMYC Q
