# Supplementary material for: Codivergence and multiple host species use by fig wasp populations of the Ficus pollination mutualism
Source: BMC Evol Biol. 2012 Jan 3;12:1. doi: 10.1186/1471-2148-12-1 (PMC3299616; doi:10.1186/1471-2148-12-1)
Supplement: Additional file 5 — Collection details of fig wasp specimens. [file 1471-2148-12-1-S5.PDF]

Additional file 5: Collection details of fig wasp specimens.

**Wasp species**	**Host fig species**	***Galoglychia* subsection**	**Collection code**	**Locality**	**Accession COI**	**Cytb**	**EF-1α**
Ceratosolen capensis	*Ficus sur*	*Sycomorus*	SA05-F27	South Africa	HM007904	HM008017	HM008130
Ceratosolen arabicus	*Ficus sycomorus*	*Sycomorus*	KN08-F62	South Africa	HM007905	HM008018	HM008131
Ceratosolen arabicus	*Ficus sycomorus*	*Sycomorus*	KN08-F58	South Africa	HM007907	HM008020	HM008133
Ceratosolen arabicus	*Ficus sycomorus*	*Sycomorus*	KN08-F56	South Africa	HM007908	HM008021	HM008134
Ceratosolen capensis	*Ficus sur*	*Sycomorus*	KN08-F01	South Africa	HM007909	HM008022	HM008135
Courtella hamifera	*Ficus ovata*	*Caulocarpae*	ZA06-F19	Zambia	HM007910	HM008023	HM008136
Courtella hamifera	*Ficus ovata*	*Caulocarpae*	ZA06-F17	Zambia	JF357988	NA	NA
Courtella armata	*Ficus sansibarica*	*Caulocarpae*	SA05-F27	South Africa	HM007911	HM008024	HM008137
Courtella armata	*Ficus sansibarica*	*Caulocarpae*	GenBank	South Africa	AY014978	NA	NA
Courtella sp.	*Ficus bizanae*	*Caulocarpae*	SA06-F100	South Africa	HM007912	HM008025	HM008138
Courtella bekiliensis	*Ficus polita*	*Caulocarpae*	KN08-F134	South Africa	HM007913	HM008026	HM008139
Courtella bekiliensis	*Ficus polita*	*Caulocarpae*	GenBank	NA	AY014977	NA	NA
Courtella bekeliensis	*Ficus polita polita*	*Caulocarpae*	SA06-F95	South Africa	JF357987	NA	NA
Courtella scobinifera	*Ficus ottoniifolia*	*Caulocarpae*	UG05-F01	Uganda	JF357989	NA	NA
Courtella sp.	*Ficus modesta*	*Caulocarpae*	MW06-F70	Mozambique	JF357985	NA	NA
Courtella sp.	*Ficus modesta*	*Caulocarpae*	MW06-F69	Mozambique	JF357986	NA	NA
Elisabethiella socotrensis	*Ficus sp. samfya ag.*	*Chlamydodorae*	ZA06-F41	Zambia	HM007915	HM008028	HM008141
Elisabethiella socotrensis	*Ficus natalensis*	*Chlamydodorae*	GenBank	South Africa	AM260706	NA	NA
Elisabethiella socotrensis	*Ficus burkei*	*Chlamydodorae*	GenBank	South Africa	AM260705	NA	NA
Elisabethiella socotrensis	*Ficus natalensis*	*Chlamydodorae*	GenBank	South Africa	AM260707	NA	NA
Elisabethiella socotrensis	*Ficus natalensis*	*Chlamydodorae*	SA05-F08	South Africa	JF357990	NA	NA
Elisabethiella socotrensis	*Ficus natalensis*	*Chlamydodorae*	SA05-F08	South Africa	JF357991	NA	NA
Elisabethiella comptoni	*Ficus abutilifolia*	*Platyphylla*	SA05-F23	South Africa	JF357992	NA	NA
Elisabethiella comptoni	*Ficus abutilifolia*	*Platyphylla*	GenBank	South Africa	AJ971652	NA	NA
Elisabethiella baijnathi	*Ficus burtt-davyi*	*Chlamydodorae*	GenBank	NA	AY014975	NA	NA
Elisabethiella baijnathi	*Ficus burtt-davyi*	*Chlamydodorae*	GenBank	South Africa	AJ971653	NA	NA
Elisabethiella stuckenbergi	*Ficus natalensis*	*Chlamydodorae*	SA05-F08	South Africa	HM007916	HM008029	HM008142
Elisabethiella stuckenbergi	*Ficus burkei*	*Chlamydodorae*	KN08-F68	South Africa	JF358004	NA	NA
Elisabethiella stuckenbergi	*Ficus lingua*	*Chlamydodorae*	MW06-F88	Mozambique	JF357999	NA	NA
Elisabethiella stuckenbergi	*Ficus burkei*	*Chlamydodorae*	GenBank	Tanzania	AM260704	NA	NA
Elisabethiella stuckenbergi	*Ficus lingua*	*Chlamydodorae*	MW06-F86	Mozambique	JF357998	NA	NA
Elisabethiella stuckenbergi	*Ficus natalensis*	*Chlamydodorae*	ZA06-F14	Zambia	JF358002	NA	NA

Elisabethiella stuckenbergi	*Ficus petersii*	*Chlamydodorae*	ZA06-F46	Zambia	JF358003	NA	NA
Elisabethiella stuckenbergi	*Ficus burkei*	*Chlamydodorae*	SA06-F98	South Africa	JF358001	NA	NA
Elisabethiella stuckenbergi	*Ficus burkei*	*Chlamydodorae*	SA05-F28	South Africa	JF358000	NA	NA
Elisabethiella stuckenbergi	*Ficus natalensis*	*Chlamydodorae*	GenBank	South Africa	AJ971651	NA	NA
Elisabethiella glumosae	*Ficus glumosa*	*Platyphylla*	KN08-F65	South Africa	HM007917	HM008030	HM008143
Elisabethiella glumosae	*Ficus glumosa*	*Platyphylla*	GenBank	NA	AY014976	NA	NA
Elisabethiella glumosae	*Ficus glumosa*	*Platyphylla*	GenBank	South Africa	AJ971654	NA	NA
Elisabethiella glumosae	*Ficus glumosa*	*Platyphylla*	SA06-F97	South Africa	JF357994	NA	NA
Elisabethiella glumosae	*Ficus glumosa*	*Platyphylla*	SA05-F19	South Africa	JF357993	NA	NA
Elisabethiella platyscapa	*Ficus fischeri*	*Chlamydodorae*	ZA06-F13	Zambia	JF357996	NA	NA
Elisabethiella sp.	*Ficus usambarensis*	*Crassicostae*	ZA06-F32	Zambia	JF357997	NA	NA
Elisabethiella sp.	*Ficus natalensis*	*Chlamydodorae*	ZA06-F14	Zambia	JF357995	NA	NA
Allotriozoon heterandromorphum	*Ficus lutea*	*Galoglychia*	SA05-F61	South Africa	JF357984	NA	NA
Alfonsiella pipithiensis	*Ficus craterostoma*	*Chlamydodorae*	KN08-F15	South Africa	HM007918	HM008031	HM008144
Alfonsiella pipithiensis	*Ficus craterostoma*	*Chlamydodorae*	KN08-F52	South Africa	HM007922	HM008035	HM008148
Alfonsiella pipithiensis	*Ficus craterostoma*	*Chlamydodorae*	GenBank	South Africa	AJ971649	NA	NA
Alfonsiella binghami	*Ficus petersii*	*Chlamydodorae*	ZA06-F46	Zambia	HM007919	HM008032	HM008145
Alfonsiella binghami	*Ficus petersii*	*Chlamydodorae*	ZA06-F46	Zambia	JF357983	NA	NA
Alfonsiella binghami	*Ficus natalensis*	*Chlamydodorae*	MW06-F89	Mozambique	HM007920	HM008033	HM008146
Alfonsiella binghami	*Ficus stuhlmannii*	*Platyphyllae*	KN08-F64	South Africa	HM007921	HM008034	HM008147
Alfonsiella binghami	*Ficus stuhlmannii*	*Platyphylla*	MW06-F60	Mozambique	JF357979	NA	NA
Alfonsiella binghami	*Ficus stuhlmannii*	*Platyphylla*	SA05-F55B	South Africa	JF357980	NA	NA
Alfonsiella binghami	*Ficus craterostoma*	*Chlamydodorae*	GenBank	South Africa	AJ971650	NA	NA
Alfonsiella binghami	*Ficus stuhlmannii*	*Platyphylla*	GenBank	South Africa	AJ971648	NA	NA
Alfonsiella binghami	*Ficus petersii*	*Chlamydodorae*	SA05-F45	South Africa	JF357981	NA	NA
Alfonsiella longiscapa	*Ficus burkei*	*Chlamydodorae*	GenBank	NA	AY014974	NA	NA
Alfonsiella longiscapa	*Ficus natalensis*	*Chlamydodorae*	MW06-F89	Mozambique	JF357982	NA	NA
Nigeriella excavata	*Ficus tettensis*	*Platyphylla*	GenBank	South Africa	AJ971655	NA	NA
Nigeriella excavata	*Ficus tettensis*	*Platyphylla*	SA05-F04	South Africa	JF358005	NA	NA
Nigeriella excavata	*Ficus tettensis*	*Platyphylla*	SA05-F31	South Africa	JF358006	NA	NA
Platyscapa soraria	*Ficus ingens*	*Urostigma*	SA05-F37	South Africa	HM007923	HM008036	HM008149
Platyscapa desertorum	*Ficus cordata*	*Urostigma*	SA07-Gg3a	South Africa	HM007924	HM008037	HM008150
