# Supplementary material for: Codivergence and multiple host species use by fig wasp populations of the Ficus pollination mutualism
Source: BMC Evol Biol. 2012 Jan 3;12:1. doi: 10.1186/1471-2148-12-1 (PMC3299616; doi:10.1186/1471-2148-12-1)
Supplement: Additional file 6 — GenBank Accession Numbers Ficus species. [file 1471-2148-12-1-S6.PDF]

Additional file 6: GenBank Accession Numbers *Ficus* species (Rønsted *et al*., 2005, 2007).

	ITS	*ETS*
F. abutilifolia	AY730091	AY730180
F. bizanae	DQ455636	DQ455670
F. burkei	AY730095	AY730184
F. burtt-davyi	DQ455647	DQ455657
F. cordata	HM746958	HM746955
F. craterostoma	AY730097	AY730186
F. glumosa	AY063562	AY063523
F. ilicina	HM746960	HM746957
F. ingens	AY730061	AY730147
F. lingua	AY730099	AY730188
F. lutea	AY063564	AY063525
F. natalensis	AY730100	AY730189
F. ottoniifolia	AY730109	AY730198
F. ovata	DQ455640	DQ455672
F. petersii	AY730101	AY730190
F. polita	DQ455642	DQ455673
F. sansibarica	AY730110	AY730199
F. stuhlmannii	AY730094	AY730183
F. sur	AY063572	AY063533
F. sycomorus	AY063575	AY063536
F. tettensis	DQ455665	DQ455683
F. trichopoda	DQ455666	DQ455684
F. umbellata	DQ455644	DQ455674
F. usambarensis	DQ455653	DQ455677
